# Development and Characterization of Electrospun Fiber-Based Poly(ethylene-*co*-vinyl Alcohol) Films of Application Interest as High-Gas-Barrier Interlayers in Food Packaging

**DOI:** 10.3390/polym13132061

**Published:** 2021-06-23

**Authors:** Beatriz Melendez-Rodriguez, Sergio Torres-Giner, Lorenzo Zavagna, Chris Sammon, Luis Cabedo, Cristina Prieto, Jose M. Lagaron

**Affiliations:** 1 Novel Materials and Nanotechnology Group, Institute of Agrochemistry and Food Technology (IATA), Spanish Council for Scientific Research (CSIC), Calle Catedrático Agustín Escardino Benllonch 7, 46980 Valencia, Spain; beatriz.melendez@iata.csic.es (B.M.-R.); storresginer@upv.es (S.T.-G.); lorenzo@zavagna.it (L.Z.); cprieto@iata.csic.es (C.P.); 2 Materials and Engineering Research Institute, Sheffield Hallam University, Sheffield S1 1WB, UK; c.sammon@shu.ac.uk; 3 Polymers and Advanced Materials Group (PIMA), School of Technology and Experimental Sciences, Universitat Jaume I (UJI), Avenida de Vicent Sos Baynat s/n, 12071 Castellón, Spain; lcabedo@uji.es

**Keywords:** EVOH, cellulose nanocrystals, electrospinning, high barrier, food packaging

## Abstract

In the present study, poly(ethylene-*co*-vinyl alcohol) with 44 mol % ethylene content (EVOH_44_) was managed to be processed, for the first time, by electrospinning assisted by the coaxial technology of solvent jacket. In addition to this, different suspensions of cellulose nanocrystals (CNCs), with contents ranging from 0.1 to 1.0 wt %, were also electrospun to obtain hybrid bio-/non-bio nanocomposites. The resultant fiber mats were thereafter optimally annealed to promote interfiber coalescence at 145 °C, below the EVOH_44_ melting point, leading to continuous transparent fiber-based films. The morphological analysis revealed the successful distribution of CNCs into EVOH_44_ up to contents of 0.5 wt %. The incorporation of CNCs into the ethylene-vinyl alcohol copolymer caused a decrease in the crystallization and melting temperatures (T_C_ and T_m_) of about 12 and 7 °C, respectively, and also crystallinity. However, the incorporation of CNCs led to enhanced thermal stability of the copolymer matrix for a nanofiller content of 1.0 wt %. Furthermore, the incorporation of 0.1 and 0.5 wt % CNCs produced increases in the tensile modulus (E) of ca. 38% and 28%, respectively, but also yielded a reduction in the elongation at break and toughness. The oxygen barrier of the hybrid nanocomposite fiber-based films decreased with increasing the CNCs content, but they were seen to remain high barrier, especially in the low relative humidity (RH) regime, i.e., at 20% RH, showing permeability values lower than 0.6 × 10^−20^ m^3^·m·m^−2^·Pa^−1^·s^−1^. In general terms, an optimal balance in physical properties was found for the hybrid copolymer composite with a CNC loading of 0.1 wt %. On the overall, the present study demonstrates the potential of annealed electrospun fiber-based high-barrier polymers, with or without CNCs, to develop novel barrier interlayers to be used as food packaging constituents.

## 1. Introduction

Polymers have been replacing the materials traditionally used in packaging, such as metal, glass, or cardboard, because they are more flexible, lighter, and habitually more cost-effective [1]. However, polymer-based materials present certain disadvantages, such as higher permeability and sorption to gases like oxygen or carbon dioxide, moisture, and organic vapors. In addition, polymer films must also present transparency, high mechanical and chemical resistance as well as be food contact approved [2]. In this sense, the thermoplastic poly(ethylene-*co*-vinyl alcohol) (EVOH), also habitually termed ethylene vinyl alcohol copolymers, are one of the most used polymer materials in high-barrier packaging films. EVOH is produced by the hydrolysis of ethylene vinyl acetate copolymer (EVA), where the acetate groups are transformed in alcohol ones [3]. In food packaging, EVOH is habitually placed in the form of thin inner layers, typically well below 10 µm, being protected from moisture by external layers of such as polypropylene (PP), polyethylene terephthalate (PET) or low- and high-density polyethylene (LDPE and HDPE) in multilayer structures [4]. The characteristics that make it suitable for this purpose are its flexibility, transparency, thermal resistance, and high-oxygen-barrier property based on its high degree of crystallinity [5,6,7]. Moreover, EVOH films are highly transparent and hydrophilic, yet water-insoluble, and can be recycled in the polyolefin regrinding process with existing infrastructure [8]. Interestingly, the EVOH copolymers, including that with 44 mol % ethylene content (EVOH_44_), with high vinyl-alcohol contents, have been proven to be able to degrade under certain environmental conditions and biological media [9,10,11,12].

The ethylene molar fraction present in the copolymer highly changes the properties of EVOH due to alterations in its molecular structure. In particular, when the ethylene content is below 42 mol %, EVOH crystals are small, dense, and in monoclinic crystal structure. However, for ethylene contents from 42 to 80 mol %, its crystals are larger, less dense, and in hexagonal crystal structure [13]. The crystallinity structure highly affects both the gas barrier and melting temperature (T_m_), being higher for the materials with lower ethylene contents, but these copolymers are also highly plasticized by moisture [14]. For instance, when the ethylene content in EVOH is increased, the oxygen transmission rate (OTR) increases exponentially [15].

In addition to multilayers, nanofillers can be employed with EVOH in order to improve their thermal, mechanical, and barrier properties. In this respect, cellulose nanomaterials have been regarded as great candidates since they are low-cost, renewable, and environmentally friendly [16]. Indeed, cellulose is the most abundant natural polysaccharide with an annual production around 75–100 billion tons [17], and it is formed of repeating rings of β-1,4-linked D-glucopyranose united by strong intermolecular hydrogen bonds [18]. It is part of the structure of plants and algae, bacteria and fungi, and tunicate [19]. There are two main types of nanocelluloses, that is, mechanically sheared cellulose nanofibers (CNFs), also termed micro-fibrillated cellulose (MFC), with amorphous and crystalline parts, and hydrolytically extracted cellulose nanocrystals (CNCs) made of high-purity single crystals [20,21]. Another type of nanocellulose derives from bacterial cellulose (BC), whose morphology can be engineered by controlling the biosynthesis pathway [22]. Among nanocellulose materials, CNC is one of the most promising nanofillers to reinforce the mechanical and barrier properties of polymers due to their high crystallinity and strong network [23]. Several studies have already reported the reinforcement achieved when CNCs have been incorporated to a polymer matrix [24,25]. For instance, a polyvinyl alcohol (PVA)/chitosan nanocomposite film reinforced with CNCs, prepared using the solvent casting and evaporation technique, showed an increase of 130% in tensile strength [26]. In other study, a PET/CNCs film improved the water vapor transmission rate of PET from 37 to 10 g·m^−2^·day^−1^ [27]. Furthermore, CNCs can serve as vehicles to develop active polymeric materials with, for instance, antimicrobial and antioxidant properties [28,29], ultraviolet light (UV) blocking [30], heavy metal absorbers [31], etc. Nanohybrids made from CNCs/metal nanoparticles (MNPs) have also gained interest due to their combined properties. Thus, oxygen scavenging nanocomposites were obtained with the incorporation of CNCs and palladium nanoparticles (PdNPs) into EVOH films [32]. CNCs acted as a support for the dispersion of the PdNPs in the polymer matrix, while the PdNPs acted as oxygen scavengers. Similarly, alginate bionanocomposite films with CNCs and silver nanoparticles (AgNPs) showed improved water and UV barrier of interest in food packaging [33].

However, there are some drawbacks to consider when CNC was used as blending element to reinforce or make more sustainable polymer matrices. In particular, CNCs tend to form aggregates in the polymer matrix causing an overall reduction of the physical properties of the nanocomposites. This agglomeration is produced by the hydrophilic nature of cellulose, which is prone to form strong intermolecular hydrogen bonds and, in the case of CNCs, this effect is increased due to its large surface area and high surface energy [34]. As a result, CNCs are prepared in the form of water suspensions, which however tend to re-agglomerate during the drying process. For instance, in spray drying, CNCs agglomeration is caused by capillary, hydrogen-bonding, and van der Waals forces. Alternatively, ice crystal growth plays a key role in CNC agglomeration during freeze drying. Agglomeration of CNCs unsuccessfully affects the mechanical and barrier advantages of the polymer nanocomposites. The minimum degree of the CNC dispersion is also so-called percolation threshold, where a three-dimensional network is obtained from a specific concentration of nanoparticles [35]. This percolation threshold depends on the aspect ratio and the orientation and distribution of the CNCs [36]. Moreover, the method used to form the nanocomposites also influences on the dispersion of CNCs [37].

Therefore, novel strategies have been explored to reduce the aggregations of CNCs prior or during their incorporation into the polymer matrices. For example, unlike spray and freeze drying, less agglomeration was reported to occur in the spray-freeze drying technique, in which the dispersed state of CNCs in water can be “frozen” in [38]. Moreover, the use of mechanical energy can be applied to separate the nanoparticles, such as high shear mixing or ultrasonication, as well as the change in the surface energy of the particles by surfactants/compatibilizers [39,40]. Traditional melt-processing methods such as extrusion [41], compounding [42], and injection molding [43] have also been used to incorporate CNCs into polymer matrices. However, the use of high temperatures during these processes could also cause the degradation of the CNCs [44]. In addition, it has been found that these methods produce more particle aggregation and mechanical degradation due to the high shear attained during processing [45]. Thus, solution processing methods such as solution casting [46], solution precipitation [47], or electrospinning [48,49,50,51,52] have been explored. In some cases, different methods can be combined to attain higher CNC dispersion [53].

In this regard, electrospinning is a promising technology for dispersing CNCs in polymer matrices since it works with polymer solutions and can incorporate the fillers into submicron fibers [54,55]. Moreover, the electrospun mats can be, thereafter, post-treated at temperatures below the T_m_ of the polymer, forming fiber-based continuous films with eliminated porosity. These materials are also called “biopapers” when made of biopolymers [56,57] due to their biofiber-based morphology and improved properties compared to traditional cellulosic papers. Thus, these materials can be produced after using minimal thermal exposure and they show good optical as well as mechanical and barrier properties, potentially offering high value in food packaging applications [58]. Moreover, electrospinning is suitable for the incorporation of nanofillers and/or functional additives within the polymer fibers, for instance volatile or thermolabile substances such as essential oils [59,60]. In addition, the resultant electrospun layers can be used as coatings or interlayers [59] to improve the mechanical and barrier properties of multilayer systems. A few previous studies have reported the electrospinning of polymers containing CNCs. For example, Redondo et al. [61] incorporated CNCs into polyurethane (PU) fibers and reported an improvement in the mechanical properties of the nanocomposite fiber mats. In another work, PVA mats prepared by electrospinning were mixed with CNC solutions to form aerogels that were subsequently hot-pressed to form nanocomposites. Authors showed good CNC dispersion with increased mechanical properties [62]. In the case of the ethylene vinyl alcohol copolymer with 27 mol % ethylene content (EVOH_27_), Martinez et al. [63] developed fibers reinforced with bacterial cellulose nanowhiskers (BCNWs) by electrospinning with a more uniform morphology than the neat EVOH_27_ fibers. However, none of the previous studies reported the production of post-processed continuous films or their properties.

The current study was aimed at obtaining for the first time (i) a new high-barrier electrospun material made of EVOH_44_ copolymer and (ii) hybrid nanocomposites made of two high-barrier materials, one of which, incorporating CNCs in powder form, can impart a stiffer and more sustainable bio-based character to EVOH_44_. The reason to select EVOH_44_ within the EVOH family is that this polymer shows lower T_m_ and, hence, lower post-processing temperatures than other EVOH family copolymers with higher vinyl-alcohol contents by which it could be more compatible with the processing temperatures of more conventional or biodegradable polymers to form multilayers. However, the processability of this copolymer by the electrohydrodynamic technique used was proven to be very difficult, so the study had to resource to the coaxial technology of solvent jacket. The study also characterized physical properties such as optical, thermal, mechanical, and barrier properties, relevant for multilayer food packaging applications of the annealed electrospun continuous films produced.

## 2. Materials and Methods

### 2.1. Materials

The ethylene vinyl alcohol copolymer grade (Soarnol AT4403) containing 44 mol % of ethylene, that is, EVOH_44_, was supplied in pellets by The Nippon Synthetic Chemical Industry Co., Ltd. (NIPPON GOHSEI, Osaka, Japan). It has a density of 1.14 g/m^3^, melt flow rate (MFR) of 3.5 g/10 min (210 °C, 2.16 kg), and a volatile content <0.3%. The CNCs were provided by CelluForce NCC^®^ (Montreal, QC, Canada). It is a 100% cellulose sulphate sodium salt, which was obtained from wood pulp. The nanofiller was supplied as a spray-dried solid white powder with a bulk density of 0.7 g/cm^3^. 2-propanol (99.5%, for analysis) was purchased by ACROS ORGANICS (Thermo Fisher Scientific, Waltham, MA, USA).

### 2.2. Preparation of Solutions 

The EVOH_44_ solutions for electrospinning were prepared in concentration of 6% (wt/vol) in a 70/30 vol/vol mixture of 2-propanol/water. The mixture was continuously stirred and heated in a thermal bath at approximately 80–90 °C on an AGIMATIC-N magnetic stirrer from JP Selecta (Barcelona, Spain). Complete dissolution of the polymer was achieved after around 2–3 h and the solutions were cooled down at room temperature prior to electrospinning. Since precipitation of the polymer always occurs after 3–4 h at room temperature [64], the precipitated mixture was heated again to 60 °C for 30–45 min when needed. Solutions containing 0.1, 0.5, and 1.0% (wt/wt) of CNCs were prepared following a similar procedure previously described [52]. Briefly, the CNC powder was first immersed in water and homogenized at 15,000 rpm for 3 min with a T25 digital Ultra-turrax from IKA^®^ (Staufen, Germany). Thereafter, the resultant dispersion was added to the polymer solution according to the compositions described above.

### 2.3. Characterization of the Solutions

All the prepared EVOH_44_ solutions, prior to electrospinning, were characterized in terms of viscosity, surface tension, and conductivity. A rotational viscosity meter Visco BasicPlus L (Fungilab S.A., San Feliu de Llobregat, Spain) with a low-viscosity adapter (LCP) was used to measure the apparent viscosity (ƞa), which was performed at 100 s^−1^. The Wilhemy plate method was followed to determine the surface tension with an EasyDyne K20 tensiometer (Krüss GmbH, Hamburg, Germany). Finally, a conductivity meter XS Con6 (Lab-box, Barcelona, Spain) was employed to evaluate the solution conductivity. Three replicates were carried out for each measurement.

### 2.4. Electrospinning Process

The electrospinning setup used consisted of an Fluidnatek^®^ LE-10 commercial lab equipment manufactured by Bioinicia S.L. (Valencia, Spain). The equipment was operated at environmental conditions of 25 °C and 40% relative humidity (RH) with a motorized single needle injector, scanning horizontally towards a metallic fixed collector to obtain homogeneous depositions. High-content vinyl-alcohol EVOH copolymers have been reported to be relatively easy to electrospin, but the one selected in this work (with 44 mol % ethylene) proved difficult to process due to very fast drying at the tip of the nozzle. To prevent needle clogging, a coaxial setup was used where pure 2-propanol was fluxed through the exterior needle to create a solvent jacket around the tip, as previously reported by Yu et al. [65]. In this setup, the sheath fluid flow-rate and the polymer solutions flow-rates were found optimal at 250 µL/h and 6 mL/h, respectively. A voltage of 24 kV and a distance between the tip and collector of 21 cm were set.

### 2.5. Film Preparation

The obtained EVOH mats were then converted into continuous fiber-based films by annealing below the polymer melting point in a 4122-model press from Carver, Inc. (Wabash, IN, USA). This post-processing was performed across the temperature range from 110 to 155 °C, for 15 s. The average thickness of all the attained films was approximately 30 µm and they were stored in a desiccator at 0% RH before characterization.

### 2.6. Characterization of the Films

#### 2.6.1. Electron Microscopy 

For the observation of the CNCs as well as fiber and film morphologies, an S-4800 scanning electron microscopy (SEM) instrument from Hitachi (Tokyo, Japan) was used. Prior to this, both the electrospun EVOH_44_ fibers and their resultant films were fixed to beveled holders using conductive double-sided adhesive tape and sputtered with a mixture of gold-palladium under vacuum. For the cross-section observations, the films were cryo-fractured by immersion in liquid nitrogen. In all cases, an accelerating voltage of 10 kV was used. The estimation of the dimensions was performed by means of the Aperture software from Apple (Cupertino, CA, USA) using a minimum of 20 SEM micrographs in their original magnification.

Transmission electron microscopy (TEM) was also performed to further study the distribution of CNCs in the EVOH_44_ fibers using a JEOL 1010 from JEOL USA, Inc. (Peabody, MA, USA) with an accelerating voltage of 100 kV. 

#### 2.6.2. Transparency

The light transmission of the films was determined using 50 mm × 30 mm specimens in an ultraviolet−visible (UV−vis) spectrophotometer VIS3000 (Dinko Instruments, Barcelona, Spain). The absorption of light was quantified at wavelengths in the 200–700 nm range. Equation (1) [66] and Equation (2) [67] were followed to determine the values of transparency (T) and opacity (O), respectively:

(1) $$T=\frac{A_{600}}{L}$$

(2) $$O={\;A}_{500}\times L$$

in which A_600_ and A_500_ correspond to the absorbance values at 600 and 500 nm, respectively, whereas L represents the film thickness (mm).

#### 2.6.3. Color

The color of the films was estimated using a Chroma Meter CR-400 (Konica Minolta, Tokyo, Japan) with the D65 illuminant. The color difference (ΔE^*^) between the samples with CNCs and the neat EVOH_44_ was determined by the Equation (3) [68]:

(3) $${\Delta E}^{*}={\left[{\left({\Delta L}^{*}\right)}^{2}+{\left({\Delta a}^{*}\right)}^{2}+{\left({\Delta b}^{*}\right)}^{2}\right]}^{0.5}$$

in which ΔL^*^ represents the difference in terms of lightness from black to white, whereas Δa^*^ and Δb^*^ correspond to the differences in color, from green to red and blue to yellow, respectively. Color changes were assessed using a previous grading: Unnoticeable (ΔE^*^  <  1), only an experienced observer can notice the difference (ΔE^* ^ ≥  1 and  < 2), an unexperienced observer notices the difference (ΔE^*^  ≥  2 and < 3.5), clear noticeable difference (ΔE^*^  ≥  3.5 and < 5), and the observer notices different colors (ΔE^*^ ≥  5) [69].

#### 2.6.4. Thermal Analysis

Thermal transitions were studied using differential scanning calorimetry (DSC) on a DSC-7 analyzer from PerkinElmer, Inc. (Waltham, MA, USA), equipped with a cooling accessory Intracooler 2 also from PerkinElmer, Inc. A two-step program, with heating and cooling rates of 10 °C/min and a nitrogen atmosphere with a flow-rate of 20 mL/min, was applied. It consisted of a first heating step from −30 to 180 °C, followed by one minute isotherm at 180 °C and a cooling run back to −30 °C. All tests were carried out in triplicate and sample weights were of ca. 3 mg. An empty aluminum pan was used as reference. Calibration was performed using an indium sample and the thermograms were corrected with those of an empty pan. The glass transition temperature (T_g_), T_m_, and enthalpy of melting (ΔH_m_) were obtained from the heating scans, while the crystallization temperature from the melt (T_c_) and enthalpy of crystallization (ΔH_c_) were determined from the cooling scans. The enthalpies were normalized to the actual polymer content in the composites. The EVOH_44_ crystallinity content (χ*_c_*) was estimated according to Equation (4) [7]:

(4) $$\chi_{c}\;\left(\%\right)=\frac{1}{\left(1-m_{f}\right)}\left[\frac{\Delta \mathrm{Hm}-\Delta \mathrm{Hcc}\;}{\Delta {\mathrm{Hm}}_{0}}\right]\times 100$$

where ∆H_m_ is the enthalpy for melting, ∆H_m0_ is melting enthalpy for a 100% crystalline EVOH sample and (1 − m_f_) is the weight fraction of EVOH_44_ in the sample. The ∆H_m__0_ value of EVOH_44_ was calculated following Equation (5) [70]:

(5) $$\Delta H_{m0}=\;\alpha \Delta H_{m0}^{\mathrm{PVA}\;}+\;\beta \Delta H_{m0}^{\mathrm{PE}\;}$$

where ∆H_m0_^PVA^ is enthalpy of melting for a 100% crystalline poly(vinyl alcohol) (PVOH), taken as 169.2 J g^−1^, and ∆H_m0_^PE^ is enthalpy of melting for a 100% crystalline of polyethylene (PE), taken as 290.0 J g^−1^, whereas α and β correspond to the weight fractions of vinyl alcohol (α = 0.56) and ethylene (β = 0.44) in EVOH_44_.

Thermogravimetric analysis (TGA) was performed in a TG-STDA model TGA/STDA851e/LF/1600 thermobalance from Mettler-Toledo, LLC (Columbus, OH, USA), under a nitrogen flow-rate of 50 mL/min. The samples, with a weight of about 15 mg, were heated from 50 to 900 °C at a heating rate of 10 °C/min. The onset degradation temperature, measured at the temperature corresponding to a 5% weight loss (T_5%_) and the thermal degradation temperature (T_deg_) were determined.

#### 2.6.5. ATR-FTIR Spectroscopy

Fourier transform infrared spectroscopy (FTIR) single spectra were collected in the 600–4000 cm^−1^ wavelength range using the Tensor 37 FTIR equipment (Bruker, Germany) coupled to the attenuated total reflection (ATR) accessory Golden Gate (Specac, Ltd., Orpington, UK). Spectra were taken by averaging 20 scans at a resolution of 4 cm^−1^.

A Nicolet Nexus FTIR instrument (Thermo Fisher Scientific, Wilmington, DE, USA) coupled to a variable-temperature single reflection diamond ATR sampling accessory (Specac Ltd., Orpington, UK) was used to collect spectra as a function of temperature. Spectra were collected using the blank ATR crystal at the same temperature as the background by averaging 64 scans at 4 cm^−1^ resolution. The samples were clamped directly onto the ATR crystal using a calibrated torque wrench (Specac Ltd.) set at 80 cNm, which applies a load of 350 N via the sample accessory anvil, to ensure that any peak intensity changes in the data represented changes to the morphology of the samples. Prior to conducting the variable temperature infrared measurements, reproducibility of the sample contact and resulting spectra intensity were validated. Spectra were collected from 30 to 130 °C at 10 °C intervals and, thereafter, up to 200 °C at 5 °C intervals. Spectra were not collected until the digital reading on the temperature controller had fully stabilized to ensure the validity of the selected temperature. 

#### 2.6.6. Mechanical Tests

The ASTM standard method D638 was followed to determine the mechanical properties of the films using an Instron 4400 universal testing machine from Instron (Norwood, MA, USA) equipped with a 1-kN load cell. Tensile tests of the films were performed with 115 × 16 mm^2^ stamped dumb-bell shaped specimens using a cross-head speed of 10 mm/min at room conditions. The samples were, prior to tensile assay, conditioned at 40% RH and 25 °C for 24 h. At least six specimens were measured for each sample.

#### 2.6.7. Permeability Tests

The water vapor permeability (WVP) of the films was determined following the standardized gravimetric method ASTM E96-95. Payne permeability cups of 3.5 cm of diameter from Elcometer Sprl (Hermallesous-Argenteau, Belgium) were used with 5 mL of distilled water. The testing was done at 25 °C on films exposed to 100% RH placed within a desiccator containing dried silica gel that generated 0% RH. Cups with aluminum films were used as control samples to estimate solvent loss through the sealing. The cups were weighted daily using an analytical balance (±0.0001 g). WVP was calculated from the regression analysis of weight loss data vs. time and the weight loss was calculated as the total loss minus the loss through the sealing. The permeability was obtained by multiplying the permeance by the film thickness. 

The oxygen permeability (OP) was determined in duplicate at 0, 20% and 80% RH and 25 °C, using an Oxygen Permeation Analyzer M8001 (Systech Illinois, Thame, UK) with temperature and RH control, purged with nitrogen before exposure to an oxygen flow of 10 mL/min. The tested area was 5 cm^2^.

### 2.7. Statistical Analysis 

The software packaging STATGRAPHICS Centurion XVI v 16.1.03 (StatPoint Technologies, Inc., Warrenton, VA, USA) was used to evaluate the differences among the samples by analysis of variance (ANOVA). Fisher’s least significant difference (LSD) was set at the 95% confidence level (*p* < 0.05).

## 3. Results and Discussion

### 3.1. Solution Properties and Morphology

The properties of the EVOH_44_ solution and its suspensions with CNCs were characterized to assess their processability by electrospinning. Table 1 summarizes the values obtained for each solution. The pure EVOH_44_ solution presented a viscosity of 71.9 cP, a surface tension of 23.8 mN/m, and a conductivity of 11.63 µS/cm. When CNCs was added to the solutions, their properties varied slightly, though the differences were still significant. Thus, for contents of 0.1, 0.5, and 1.0 wt % of CNCs in the EVOH_44_ solutions, the values for viscosity, surface tension, and conductivity were in the range of 74–80 cP, 24–25 mN/m, and 11.8–12.1 µS/cm. The slight increase in viscosity can be related to the presence of the nanofiller in the suspensions, which could stablish secondary bonding interactions with the EVOH_44_ molecules via the hydroxyl groups. Similarly, the slight increase in surface tension and conductivity may be related to the higher polarity of the nanocellulose particles. Changes in the electrospun morphologies of EVOH due to variations in solution properties have been previously studied [71]. In particular, a decrease in the diameter of EVOH fibers was reported when the solution conductivity decreased.

**Figure 1:**
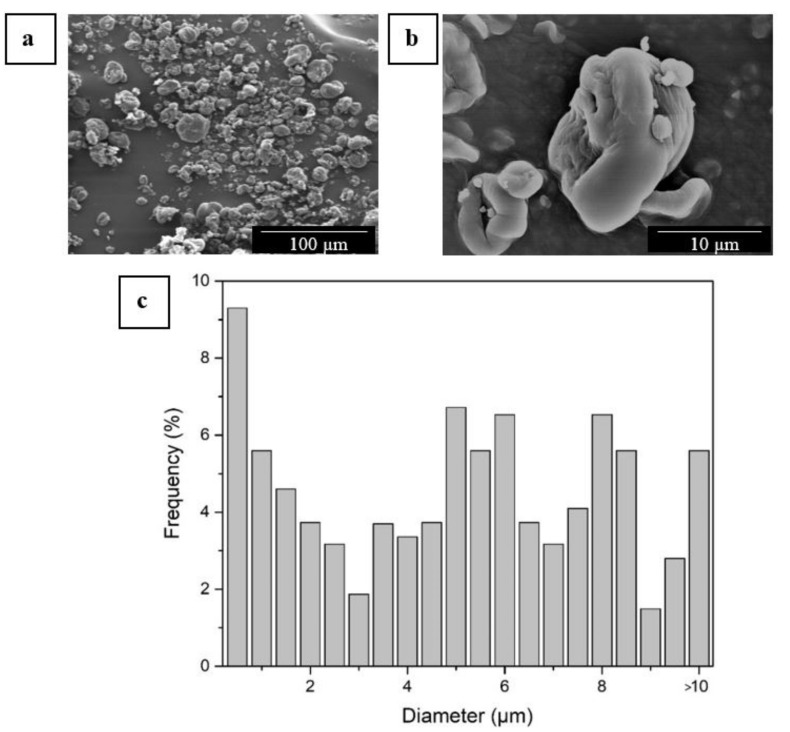
Scanning electron microscopy (SEM) images of the as-received cellulose nanocrystals (CNCs) in powder form taken at low (**a**) and high (**b**) magnification, showing scale markers of 100 and 10 μm, respectively; (**c**) Diameter histogram.

The morphology of the as-received CNC powder was observed by SEM. Figure 1 shows the SEM micrographs of the CNCs at both low and high magnification, that is, 400× (Figure 1a) and 3000× (Figure 1b), respectively. It can be observed that CNCs were mainly in the form of shrunken particles with a wide particle size distribution (Figure 1c). This particular type of morphology is known to occur during spray-drying due to the rapid evaporation of the solvent and the formation of an external crust during the first stages of drying, which collapses when the solvent present in the inner parts of the droplet evaporates and leads to a partial shrinkage of the particle [72]. It can also be observed that particle sizes varied from large particles of nearly 20 µm down to nanoparticles below 100 nm. A similar morphology, showing the co-presence of nanoparticles and large particles as a result of agglomeration has been previously reported for CNCs processed by spray-drying [73,74,75]. Agglomeration is produced during the drying process when the capillary, van der Waals, and hydrogen bonding forces overcome the electrostatic repulsion force produced by the negative charge on the surface of the CNCs [76]. The resulting powdered product obtained by the spray-drying technique, thus, consists of compact particles typically in the micro-size range.

**Figure 2:**
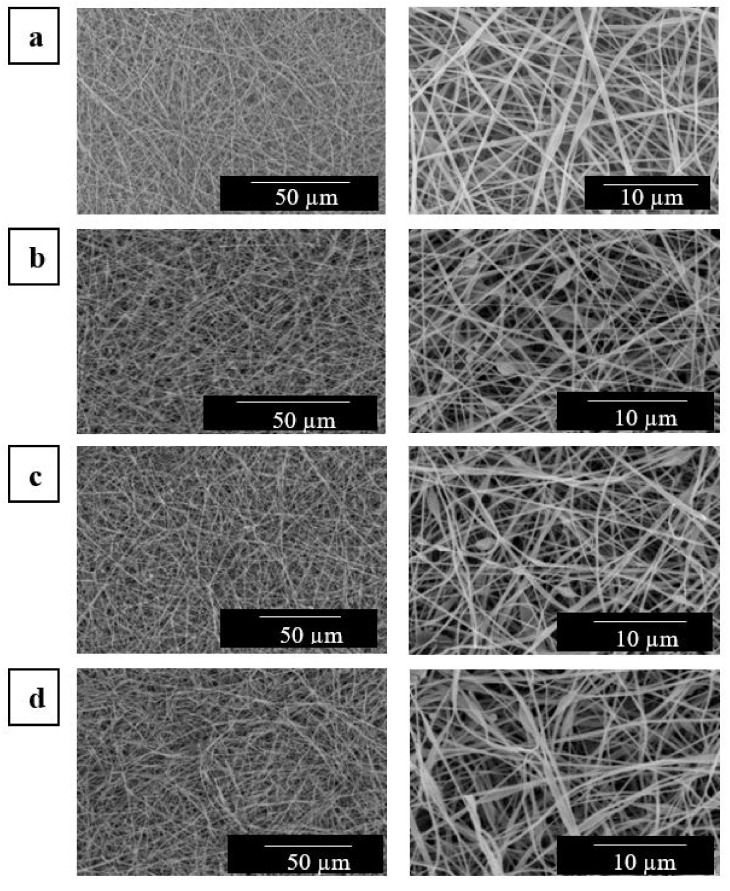
Scanning electron microscopy (SEM) images of the electrospun mats of poly(ethylene-*co*-vinyl alcohol) with 44 mol % of ethylene (EVOH_44_), pure (**a**) and with cellulose nanocrystals (CNCs): (**b**) 0.1 wt %; (**c**) 0.5 wt %; (**d**) 1.0 wt %. Left images were taken 800× at with scale markers of 50 μm, while right images were taken at 3000× with scale markers of 10 μm.

The SEM images of the electrospun mats of the EVOH_44_ fibers, prior to the thermal post-treatment, are gathered in Figure 2. As it can be seen from these micrographs, all the mats, even those of neat EVOH_44_, presented some beads along the fiber axis. The beaded fiber morphology observed herein is similar to those observed in previous works reporting the preparation of electrospun fibers of EVOH with different contents of ethylene [63,77]. The presence of beads in the fibers are habitually associated to a non-optimal concentration of the polymer and/or of the operating parameters [78]. However, the concentration and parameters used resulted in successful electrospinning for this material. The mean diameters of the beaded regions increased with the CNCs content, in a range from 1.1 to 1.8 µm. As shown in previous Table 1, the mean diameter of the neat EVOH_44_ fibers obtained by electrospinning, included in Figure 2a, were around 410 nm. The incorporation of CNCs resulted in an increase in the diameter of the EVOH_44_ fibers for contents above 0.5 wt %. Thus, the samples with a 0.1 wt % CNCs (see Figure 2b) exhibited fibers with similar size as the pure EVOH_44_, that is, 410 nm, but with more beaded regions. In the case of the samples with 0.5 and 1.0 wt % of CNCs, gathered respectively in Figure 2c,d, the fibers showed mean diameters of approximately 502 and 592 nm. This effect can be ascribed to the slight viscosity increase described above. This result differs from previous studies in which the presence of CNCs reduced fiber diameter due to an increase in conductivity as CNCs is negatively charged [79,80]. Moreover, the bigger beaded areas in the fibers could be generated to accommodate CNCs agglomerates.

**Figure 3:**
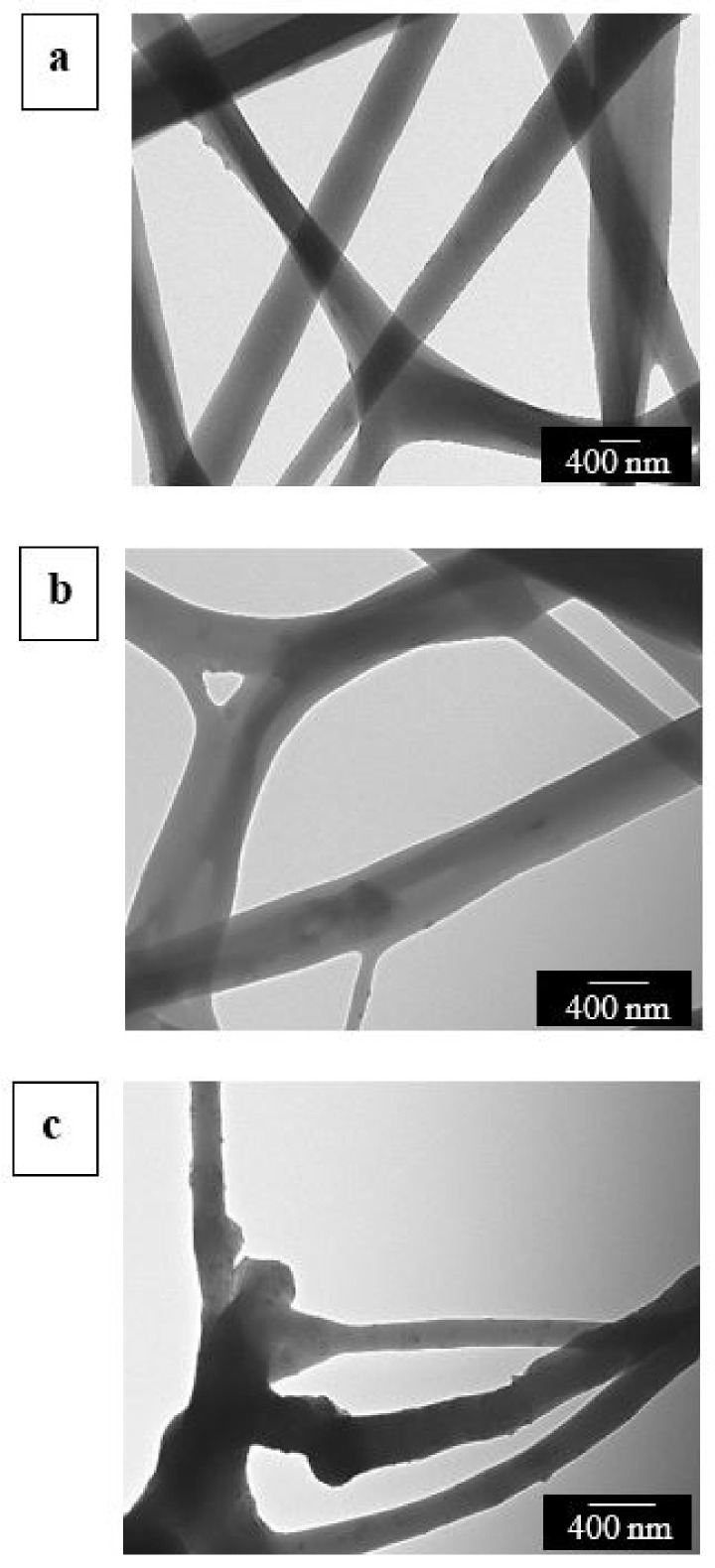
Transmission electron microscopy (TEM) images of the electrospun fibers of poly(ethylene-*co*-vinyl alcohol) with 44 mol % of ethylene (EVOH_44_) with cellulose nanocrystals (CNCs): (**a**) 0.1 wt %; (**b**) 0.5 wt %; (**c**) 1.0 wt %. Images taken at 10,000× with scale markers of 400 nm.

As a result of potential agglomeration of CNCs in the electrospun EVOH_44_ fiber beaded regions, the fibers were also observed by TEM. Figure 3 displays the TEM micrographs of the electrospun fibers, in which it can be discerned that the CNC distribution was highly dependent on the added content. For electrospun fibers with a content of 0.1 wt %, shown in Figure 3a, the incorporated CNCs were relatively well distributed along the fiber axis since agglomerates are hard to spot. Figure 3b reveals that the electrospun EVOH_44_ fibers with 0.5 wt % also showed a good distribution of CNCs. However, agglomeration was very noticeable in Figure 3c, in which it can be observed that CNCs at 1.0 wt % were mainly located in the beaded regions. Similar results have been observed previously for BCNWs incorporated into polyethylene oxide (PEO) fibers by electrospinning [81].

### 3.2. Thermal Properties of the Electrospun Fibers

**Figure 4:**
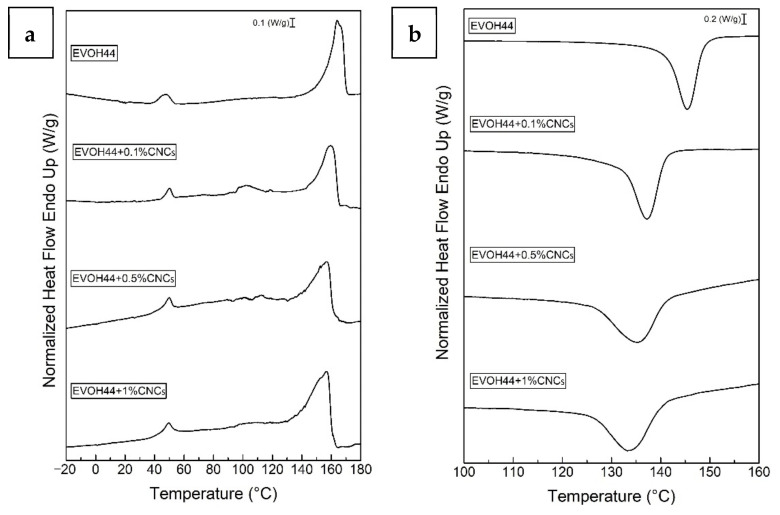
Differential scanning calorimetry (DSC) curves during first heating (**a**) and cooling (**b**) of the electrospun fibers of poly(ethylene-*co*-vinyl alcohol) with 44 mol % of ethylene (EVOH_44_) with and without cellulose nanocrystals (CNCs).

The DSC curves for the different electrospun EVOH_44_ fiber mats of the various materials, corresponding to the first heating and cooling steps, are gathered in Figure 4. 

Table 2 displays the main thermal values obtained from the DSC curves. Regarding the neat EVOH_44_, one can observe in Figure 4a that, during the first heating, it showed a T_g_ at approximately 41 °C. The low-intensity endothermic peak observed in the glass transition region is often connected with endothermic relaxation phenomena occurring at the transient state from glassy to rubbery for the amorphous phase. Then, the EVOH_44_ copolymer melted at nearly 164 °C with a ΔH_m_ of 60.1 J/g, resulting in a crystallinity degree of 27.0%. In relation to the EVOH_44_ composites, the T_g_ values increased in temperature, ranging between 41.7–44.0 °C, suggesting a filler-induced “rigidization” of the amorphous phase. This effect was seen maximum for the sample with the highest dispersed amount of CNCs, that is, 1 wt %. The fibers of the nanocomposites also exhibited a somewhat broader melting endothermic events towards lower temperatures than the pristine polymer. Thus, the melting points and enthalpies were seen to decrease with increasing filler content, suggesting a crystallinity distortion induced by the filler. More specifically, the T_m_ of EVOH_44_ was reduced by 7.1 °C in the nanocomposite with 1 wt % CNCs, and the crystallinity decreased to 23.4%, 24.1%, and 22.8% for the 0.1, 0.5, and 1.0 wt % CNC content, respectively. In this regard, it is interesting to mention that Martínez-Sanz et al. [52] also reported a decrease in T_m_ by adding BCNWs to electrospun EVOH_29_ fibers. Alterations in crystallinity have been linked to a covalent or hydrogen bonding between the EVOH hydroxyl groups (OH) and low-molecular weight (M_W_) additives in the amorphous phase [82].

Figure 4b also shows that in agreement with the melting data, the neat EVOH_44_ showed during crystallization a T_c_ value centered at 145.4 °C and a ΔH_c_ value of 61.8 J/g, that decreased and broadened with increasing the filler content. In particular, the lowest T_c_ value was observed for the nanocomposite sample with 1 wt % CNCs, with a reduction of 12.2 °C. Thus, the presence of CNCs impedes the proper lateral order of the copolymer chains, requiring higher undercoolings to crystallize, and doing so to a lesser extent than in the unfilled material.

The results obtained for the here-prepared electrospun EVOH_44_ fibers agree reasonably well with those reported in the literature for materials produced by other techniques. For instance, EVOH_44_ films obtained by extrusion showed T_m_ values around 160 °C and 162 °C, during the first and second heating, respectively [83]. In another study, EVOH_44_ films prepared by solvent casting presented a T_c_ of 140 °C and T_m_ values of 163 °C and 165 °C, during the first and second heating, respectively [84]. Moreover, in the latter study, MFC was added to solvent-cast EVOH_44_ films, reporting also lower T_c_ and T_m_ values compared to the pristine copolymer.

### 3.3. Film Forming by Interfiber Coalescence Induced by Annealing 

**Figure 5:**
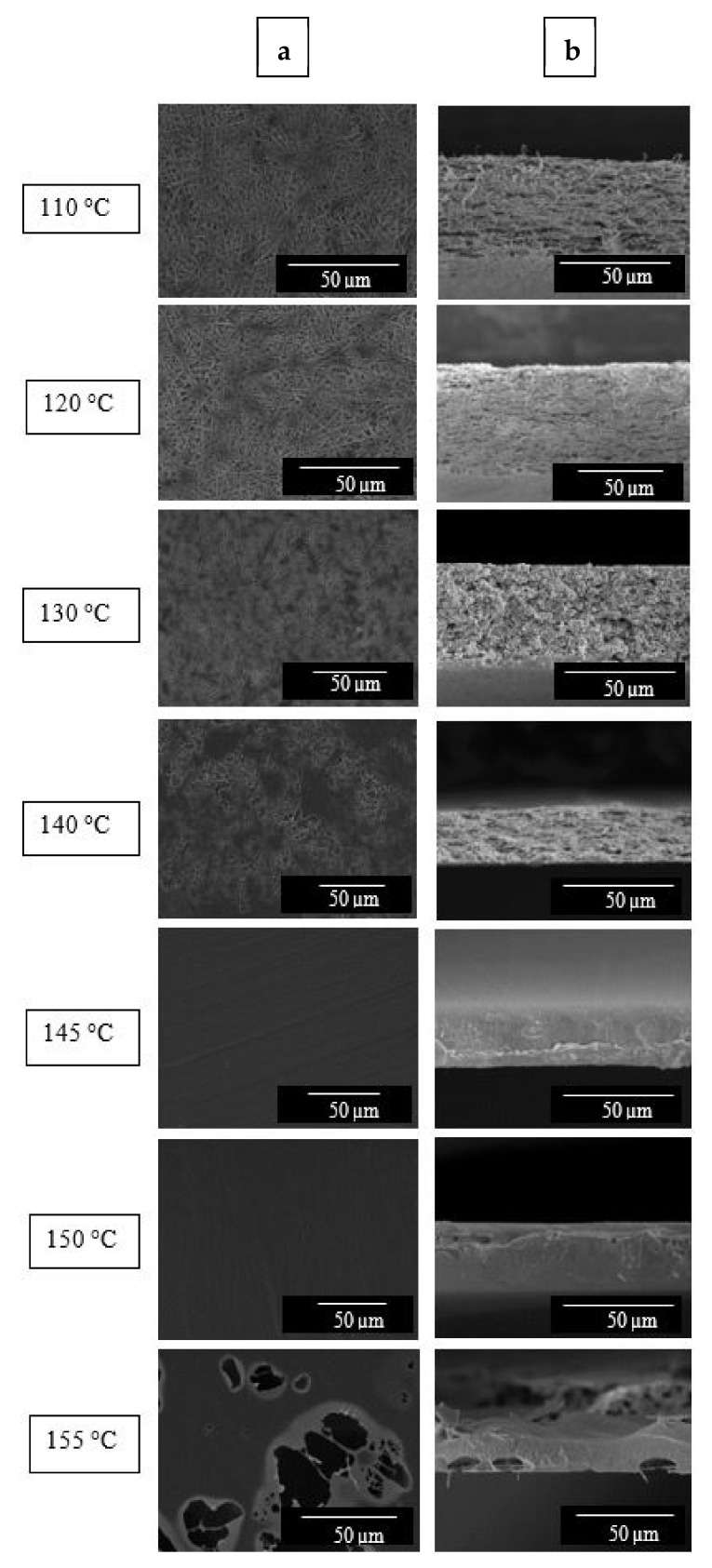
Scanning electron microscopy (SEM) images at the top view (**a**) and cross-section (**b**) of the electrospun mats of poly(ethylene-*co*-vinyl alcohol) with 44 mol % of ethylene (EVOH_44_) annealed at: 110, 120, 130, 140, 145, 150, and 155 °C for 15 s. Images were taken at 1100× with scale markers of 50 μm.

In order to induce fiber mat densification, different annealing temperatures below the copolymer melting point were screened to ascertain the most optimal thermal treatment that can lead to continuous fiber-based films better suited for their use in packaging. The morphology evolution of the electrospun materials obtained at different annealing temperatures is shown in Figure 5 from top views (Figure 5a) and cryo-fracture surfaces (Figure 5b). From this figure, it can be seen that at temperatures below 145 °C, the electrospun materials still showed some level of porosity and non-homogeneous surface. At 145 °C and 150 °C, a compact interfiber coalescence of the EVOH_44_ fibers occurred, causing a material densification and alignment side by side of the fibers. At 155 °C, the continuous structure was lost due to the appearance of holes caused by partial melting of the copolymer. As a result, 145 °C was selected as the minimum annealing temperature, below the melting point of EVOH_44_, to thermally process the material to turn it into a continuous film. 

**Figure 6:**
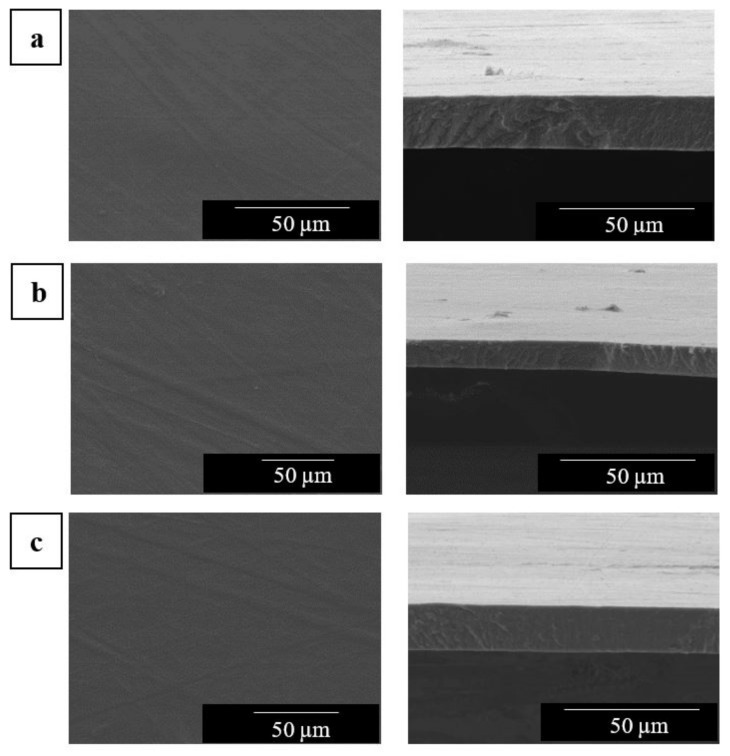
Scanning electron microscopy (SEM) images in top view (left) and cross-section (right) of the electrospun mats of poly(ethylene-*co*-vinyl alcohol) with 44 mol % of ethylene (EVOH_44_) with cellulose nanocrystals (CNCs): (**a**) 0.1 wt %; (**b**) 0.5 wt %; (**c**) 1.0 wt %. The electrospun mats were thermally post-treated at 145 °C for 15 s. Images were taken at 1100× with scale markers are of 50 μm.

Similarly, the electrospun mats of EVOH_44_ with the different CNC contents were also annealed at 145 °C and, thereafter, observed by SEM. The resulting morphology in both top view and cross-section of the materials, are presented in Figure 6. As it can be seen, homogeneous fiber-based films were obtained in all cases.

**Figure 7:**
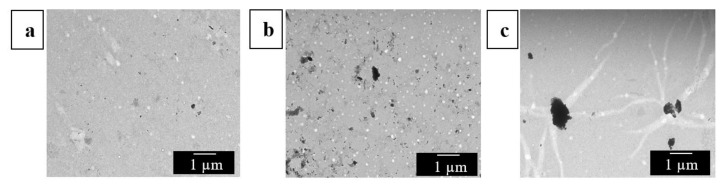
Transmission electron microscopy (TEM) images of the annealed electrospun films of poly(ethylene-*co*-vinyl alcohol) with 44 mol % of ethylene (EVOH_44_) with cellulose nanocrystals (CNCs): (**a**) 0.1 wt %; (**b**) 0.5 wt %; (**c**) 1.0 wt %. Images were taken at 3000× with scale markers of 1 µm.

Furthermore, the distribution of the CNCs in the electrospun biopapers of EVOH_44_ was also analyzed by TEM. In Figure 7, it can be seen that, as was previously observed in the electrospun fibers, the CNC distribution was better at both 0.1 wt % (Figure 7a) and 0.5 wt % loadings (Figure 7b), whereas more nanocrystal aggregates were easily found at the highest content, that is, at 1.0 wt % (Figure 7c). These results suggest that the CNC distribution achieved in the EVOH_44_ fibers during electrospinning was seemingly preserved in the films. 

### 3.4. Variable-Temperature FTIR Spectroscopy

**Figure 8:**
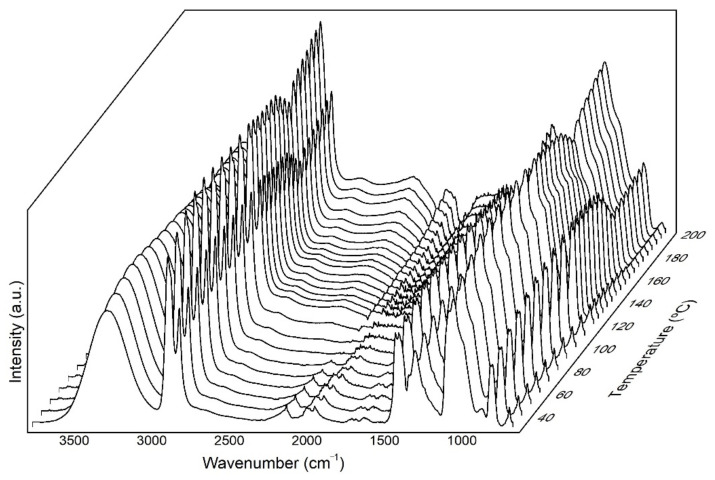
Fourier transform infrared spectroscopy (FTIR) spectra taken during heating of the electrospun fibers of poly(ethylene-*co*-vinyl alcohol) with 44 mol % of ethylene (EVOH_44_).

An insight into the spectral changes associated to the molecular order of the electrospun EVOH_44_ fibers was carried out by ATR-FTIR during heating from 30 to 200 °C, as it can be seen in Figure 8.

One characteristic feature of the EVOH spectra is the peak centered at approximately 3330 cm^−1^, which corresponds to the stretching band of the O-H oscillators [85]. The breadth and position of this band indicates the presence of strong hydrogen bonding within the copolymer, the broadness is due to intra- and intermolecular hydrogen-bonded OH dimer and multimers, with varying strengths and geometries [86,87]. In addition, the EVOH_44_ spectra showed bands at nearly 2933 and 2852 cm^−1^ corresponding to the C-H antisymmetric and symmetric stretching vibrations, respectively. The features at approximately 1437 and 1456 cm^−1^ are attributed to OH deformation, the band at 1374 cm^−1^ is likely arising from OH deformation, and CH_2_ wagging and the band at 842 cm^−1^ is assigned to skeletal vibrations and CH_2_ rocking [88,89]. 

Many FTIR bands in polymers are conformationally sensitive, being the sharper peaks associated to ordered chain segments along the backbone, arising mostly from within crystals. When the relative intensity of conformationally sensitive bands rises upon heating, they typically sharpen, shifting in position and become narrower [57]. From Figure 8, a progressive increase in the intensity of many bands was initially observed, associated to classical crystal perfecting, followed by a leveling-off of these and a subsequent decrease up to around 160 °C. The steeper final drop in intensity observed is ascribed to the decrease in molecular order preceding to the melting of the sample. Therefore, at the selected annealing temperature of 145 °C, many of the bands in the spectra are in the regime in which they begin to decrease intensity, suggesting that the molecular order has started to decreased. Therefore, at this temperature, enough thermally-induced molecular motions are enabled for the interfiber coalescence process to occur, being able to reduce the high surface energy of the fibers.

### 3.5. Optical Properties of the Annealed Electrospun Films

**Figure 9:**
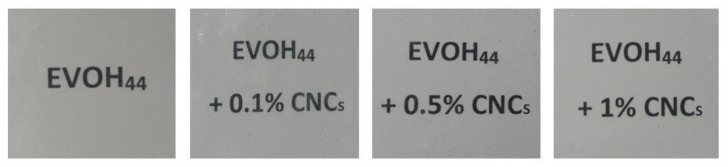
Contact transparency pictures of the annealed electrospun films of poly(ethylene-*co*-vinyl alcohol) with 44 mol % of ethylene (EVOH_44_) with and without cellulose nanocrystals (CNCs).

The contact transparency pictures of the films are gathered in Figure 9. From this figure, it can be seen that, regardless of the CNC content, all the EVOH_44_ film samples presented high contact transparency. Similar good optical properties have been reported earlier for electrospun fibers subjected to interfiber coalescence by annealing [71,77]. Table 3 shows that the color parameters changed slightly between the different samples, in most cases being not significant, showing values ranging between 2.79–2.84, −4.69−(−5.10), and 90.84–91.28, for the a^*^, b^*^, and L^*^, respectively. This implies that all the films were luminous with a slight tendency towards red and blue colors. In terms of color difference, the film samples with CNCs showed a ΔE^*^ value < 1, which indicates that the color change with respect to the neat EVOH_44_ film was unnoticeable. On the other hand, regarding transparency, it was observed that the T and O values decreased with increasing CNC content. Thus, the neat EVOH_44_ film and the composite film with 0.1 wt % CNCs showed T values of 7.9 and 7.7, respectively. However, EVOH_44_ with 0.5 and 1.0 wt % CNCs presented T values of 4.6 and 3.6, respectively. In terms of opacity, the O values slightly decreased from 0.004 to 0.002. Since the color variations were minimal and the nanocomposite films preserved most of the high transparency of the EVOH_44_ film, it is inferred again the good distribution of the dispersed nanofillers across the copolymer matrix. Similar results were reported by Martinez-Sanz, after the incorporation of BCNWs into an EVOH_29_ matrix by electrospinning before melt-mixing. In the previous work, it was already demonstrated that this methodology can be an efficient process to disperse BCNWs, since the resultant nanocomposites exhibited higher transparency than the ones developed by direct melt-mixing [52]. 

### 3.6. Thermal Stability of the Annealed Electrospun Films

**Figure 10:**
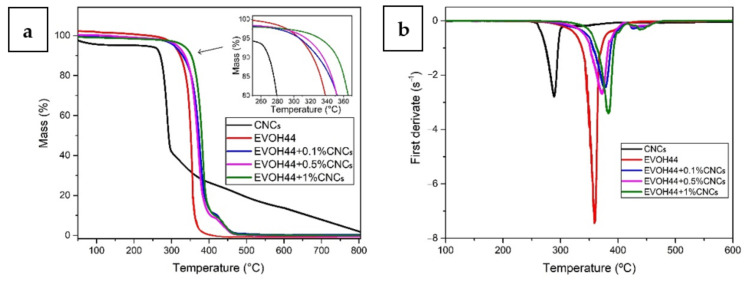
(**a**) Thermogravimetric (TGA) and (**b**) first derivative (DTG) curves of the cellulose nanocrystals (CNCs) and of the annealed electrospun films of poly(ethylene-*co*-vinyl alcohol) with 44 mol % of ethylene (EVOH_44_) with and without CNCs.

Figure 10 shows the TGA curves of the CNC powder and electrospun EVOH_44_ films, whereas Table 4 gathers the main TGA parameters. The CNCs presented a T_5%_ at 215.6 °C and a T_deg_ at 288.9 °C with a mass loss of 41.3% and a residual mass of 5.8%. These results are consistent with the existing literature for CNCs. For example, Mano et al. [90] showed that the main thermal degradation of CNCs occurred at about 277 °C. Similar results were reported by Cheng et al. [91], where CNCs presented a T_deg_ close to 300 °C.

In Figure 10 and Table 4 it can be seen that the electrospun neat EVOH_44_ film showed a T_5%_ and a T_deg_ at 306.8 °C and 359.7 °C, respectively, with a mass loss of 48.5% and a residual mass of 0.1%. These results are consistent with other values reported in the literature. For instance, a bar-coated film of poly(ethylene-co-vinyl alcohol) with 32 mol % of ethylene (EVOH_32_) film showed a T_deg_ at 349.1 °C [92]. In another study, a solvent-casted EVOH_32_ film presented a T_5%_ and a T_deg_ at 270.5 °C and 381.0 °C, respectively, and a residual mass of 2.2% [32]. The addition of CNCs improved the thermal stability of EVOH_44_ since a clear increase in both T_5%_ and a T_deg_ was observed, particularly for contents above 0.5 wt %. Thus, the T_5%_ increased to 314.9 °C and 331.2 °C, for the EVOH_44_ films with a 0.5 wt % and 1 wt % CNCs, respectively. The T_deg_ values also increased, being located at 377.4, 372.6, and 383.6 °C, for the film samples with 0.1, 0.5, and 1.0 wt % CNCs, respectively. Finally, all the nanocomposite films presented a mass loss at T_deg_ of approximately 60% and a residual mass of 0.1%. Therefore, CNCs successfully delayed the thermal degradation of EVOH_44_. Furthermore, the nanofillers also benefited from EVOH_44_ since they seemed to be better stabilized in the copolymer matrix. This phenomenon has been previously reported by Orr et al. [93], who prepared and characterized EVOH_48_ films with CNCs by the solution casting method. Also, Noorani et al. [94] reported an increase in thermal stability when CNCs was added to a polysulfone resin by a solvent exchange process, indicating not only good CNCs dispersion, but also a good interfacial interaction with the polymer matrix.

### 3.7. ATR-FTIR Spectroscopy of the Annealed Electrospun Films

**Figure 11:**
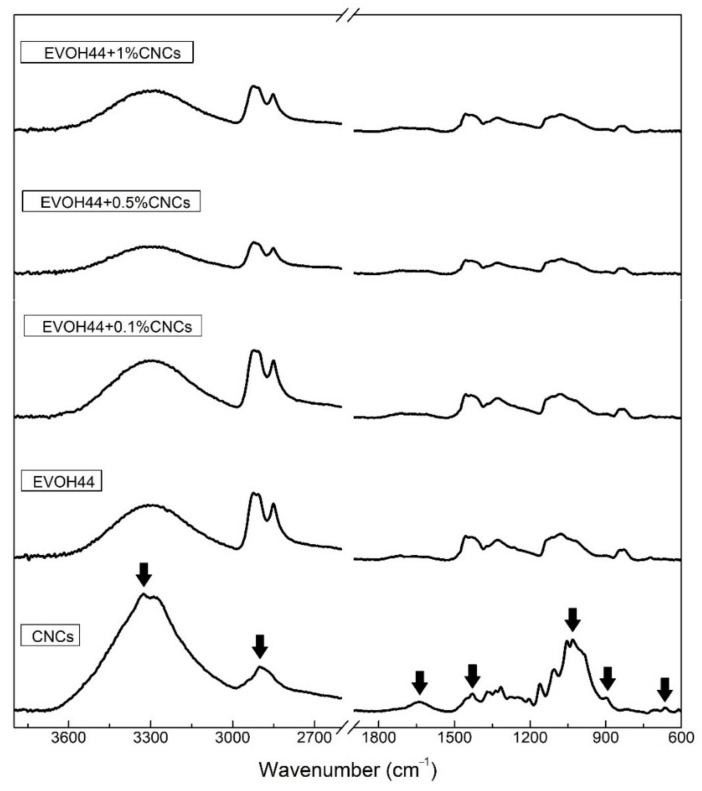
Fourier transform infrared spectroscopy (FTIR) spectra of the cellulose nanocrystals (CNCs) powder and of the annealed electrospun films of poly(ethylene-*co*-vinyl alcohol) with 44 mol % of ethylene (EVOH_44_) with and without CNCs. The arrows refer to the bands discussed in the text.

Figure 11 displays the FTIR spectra of the CNC powder and of the various electrospun EVOH_44_ mats loaded with CNCs. For the CNCs powder, the strong and broad absorption peak at 3326 cm^−1^ was ascribed to the −OH stretching vibration of the sample with a contribution from any sorbed water [95]. Assignments can also be made for the peak at 2900 cm^−1^ (CH stretching vibrations), 1054, and 898 cm^−1^ (C-O stretching) [96,97,98]. The band at 1640 cm^−1^ was also associated with the sorbed water [97]. Furthermore, the peaks at 1612 and 1429 cm^−1^ were assigned to asymmetric and symmetric stretching vibrations of −COOH [99]. Finally, the 667 cm^−1^ peak was attributed to the C–OH out-of-plane bending mode [100]. There was no indication of remaining lignin in the CNCs since the lignin contribution has characteristic peaks around 1500 cm^−1^ [101]. 

Upon the addition of CNCs to the pure EVOH_44_, there was no evidence of any new peaks or changes in the position of the characteristic EVOH_44_ peaks. This result suggests that, for the loadings used, there was no detectable interaction that could be picked up by the technique between EVOH_44_ and CNCs, which are both known to be strongly self-associated polymers. These finding are in accordance with those reported elsewhere in the literature for other similar systems. For example, in an EVOH_32_ film with BCNWs, the cellulose characteristic bands were not observed up to concentrations of 3 wt % BCNWs [77]. In another study, an EVOH_32_ film with 1.0 wt % CNCs was also no loaded enough to show unambiguous changes in the spectra [32]. 

### 3.8. Mechanical Properties of the Annealed Electrospun Films

**Figure 12:**
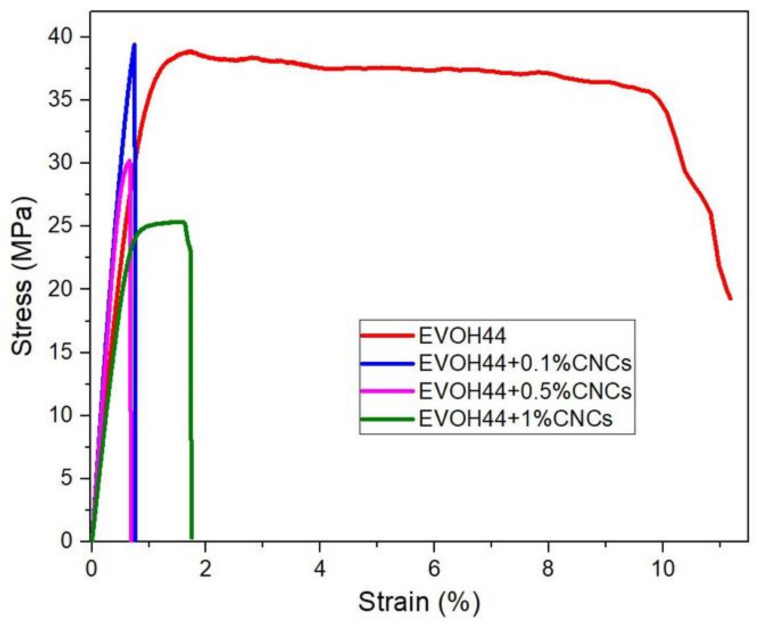
Tensile stress–strain curves of the annealed electrospun films of poly(ethylene-*co*-vinyl alcohol) with 44 mol % of ethylene (EVOH_44_) with and without cellulose nanocrystals (CNCs).

Table 5 gathers the values of the elastic modulus (E), tensile strength at break (σ_b_), elongation at break (ε_b_), and toughness (T) of the electrospun EVOH_44_ films calculated from their strain-stress curves obtained at room temperature and shown in Figure 12. The neat EVOH_44_ film showed values of E of 4699 MPa, σ_y_ of 38.7 MPa, ε_b_ of 10.4%, and T of 3.2 mJ/m^3^. When CNCs were incorporated into EVOH_44_, the E values increased for contents of 0.1 and 0.5 wt %, by 37.75% and 28.15%, respectively, while σ_y_ was kept in the 30–40 MPa range, hence resulting in more rigid films. However, the mechanical strength decreased at the CNC loading of 1.0 wt %, showing values of E and σ_y_ of 4135 and 25.2 MPa, respectively. Moreover, the ductility of the electrospun EVOH_44_ films decreased significantly for all the CNC contents, showing values of ε_b_ and T in the ranges of 0.7–1.6% and a T of 0.2–0.3 mJ/m^3^.

Previous works dealing with polymer nanocomposites based on CNCs have reported a similar mechanical behavior. For instance, EVOH_32_/cellulose nanowhisker (CNW) nanocomposites prepared by melt compounding showed an increase in tensile modulus and tensile strength but accompanied with a decrease in ductility when increasing the nanofiller content, producing stronger but more brittle materials [102]. Also, polylactide acid (PLA) nanocomposite fibers containing 1–3 wt % BCNWs prepared by electrospinning and, then, melt-mixed with PLA pellets by melt compounding showed a percentage increase in Young’s modulus and tensile strength of about 15%, but a decrease in ε_b_ of 10% [103]. In another study, PLA/MFC nanocomposites prepared by solvent mixing and, then, hot-pressed into sheets, also presented an increase in both tensile modulus and tensile strength, but a decrease in strain at break [104]. Similarly, functionalized cellulose nanocrystal methyl ester (CNC-me) incorporated into PHBV films prepared by solution casting showed an increase in Young’s modulus and tensile strength of 250% and 147%, respectively, when increasing the CNC content, while ε_b_ considerably decreased [105]. A similar effect was observed for PVOH films reinforced with CNCs also prepared by solvent casting [106,107]. Different factors have been hypothesized to influence the mechanical properties of polymers reinforced with CNCs. Thus, in addition to potential interactions between the polymer matrix and cellulosic nanofillers in the amorphous phase, the dispersion and distribution of the CNCs in the matrix has a significant effect [105]. In particular, it has been described that an increase in mechanical strength is due to a good stress transfer across the interphase due to the interfacial bond that occurs between the CNCs and the polymer matrix [108,109]. Moreover, achieving the percolation threshold is critical for obtaining an enhanced mechanical performance. This consists on the formation of a 3-dimensional (3D) nanocrystal network via hydrogen bonding forces that connect the fillers throughout the polymer matrix. This phenomenon can be affected by different parameters, such as particle interactions, orientation, or aspect ratio [110]. With CNCs, it has been described that a good dispersion of the nanocrystals, without agglomerations, can favor the elimination of the defects or stress concentrators [39]. Therefore, even though, the FTIR analysis did not resolve any potential hydrogen bonding interactions between the components, it is clear that the electrospun EVOH_44_ films having 0.1 wt % CNCs, with best reported filler distribution, showed an optimal balance in mechanical properties. On the contrary, the sample with 1.0 wt % CNCs, which showed more agglomerations, presented a reduction in the reinforcement of the copolymer matrix.

### 3.9. Barrier Properties of the Annealed Electrospun Films

**Figure 13:**
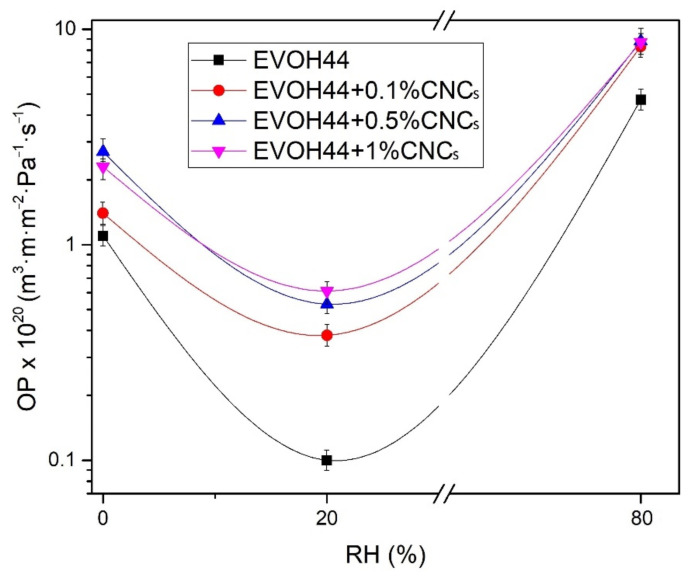
Evolution of oxygen permeability (OP) in log scale as a function of the percentage of relative humidity (% RH) of the annealed electrospun films of poly(ethylene-*co*-vinyl alcohol) with 44 mol % of ethylene (EVOH_44_) with and without cellulose nanocrystals (CNCs).

The WVP and OP values of the annealed electrospun EVOH_44_ films, with and without CNCs, are shown in Table 6. The neat EVOH_44_ film showed the highest barrier performance, that is, the lowest permeabilities. Thus, for WVP, EVOH_44_ film showed a value of 1.6 × 10^−14^ kg·m·m^−2^·Pa^−1^·s^−1^. OP was measured at different % RH, namely 0%, 20%, and 80%, in order to assess the effect of humidity on the oxygen permeability. From Figure 13, which plots the evolution of the oxygen barrier as function of % RH, it can be observed that the EVOH_44_ film presented good oxygen barrier at low humidity, showing values of 1.1 × 10^−20^ and 0.1 × 10^−20^ m^3^·m·m^−2^·Pa^−1^·s^−1^ for 0% and 20% RH, respectively. However, at the highest humidity, that is, 80%, the barrier decreased to 4.7 × 10^−20^ m^3^·m·m^−2^·Pa^−1^·s^−1^. In hydrophilic polymers, which is the case of the EVOH copolymers, it has been reported an increase in the permeability to oxygen gas due to water-induced plasticization at high humidity [111]. In this plasticization regime, sorbed water molecules intercept the strong polymer interchain self-association, leading to water molecules clustering, and hence to an increase in free volume that allow the gas molecules to diffuse through [89]. It is also known that at lower humidity, the sorbed water is not able to break the strong interchain hydrogen bonding, hence the water molecules block the existing free volume instead, thus reducing the available sites for diffusion [111]. 

Compared to other EVOH_44_ films studied in the literature, the values reported herein for the EVOH_44_ films are within the same order of magnitude. For instance, EVOH_44_ films prepared by extrusion showed a WVP of 0.25 × 10^−14^ kg·m·m^−2^·Pa^−1^·s^−1^ [103], whereas for others prepared by solvent casting it was 0.11 × 10^−14^ kg·m·m^−2^·Pa^−1^·s^−1^ [84]. In terms of oxygen barrier, an OP value of 0.77 × 10^−21^ m^3^·m·m^−2^·Pa^−1^·s^−1^ was reported for EVOH_32_ in dry conditions, while a value of 9.1 × 10^−20^ m^3^·m·m^−2^·Pa^−1^·s^−1^ was obtained in wet conditions [112]. The OP of a solvent-cast EVOH_44_ film was studied at different % RH, resulting in values of 0.42 × 10^−20^ m^3^·m·m^−2^·Pa^−1^·s^−1^ at 65% RH and 0.26 × 10^−20^ m^3^·m·m^−2^·Pa^−1^·s^−1^ at 0% RH [113]. Also, the OP of a melt-extruded EVOH_44_ film was measured at different % RH, with values of 9 × 10^−20^ m^3^·m·m^−2^·Pa^−1^·s^−1^ at 50% RH and 9 × 10^−19^ m^3^·m·m^−2^·Pa^−1^·s^−1^ at 90% RH. These results indicate that the barrier properties of the annealed electrospun fiber-based EVOH_44_ films obtained are somewhat lower, but within the same order of magnitude, than films of this copolymer processed by other techniques. 

When CNCs were incorporated into the electrospun EVOH_44_ fibers, an increase in permeability was observed in the resulting films. In general terms, the films with different CNC contents showed minor differences between them, in most cases being not significant, though there was a tendency for the barrier properties to decrease with increasing the nanofiller content. Finally, the OP values were also determined at the three % RH tested, exhibiting a similar trend as for the case of the EVOH_44_ film. Therefore, the best barrier performance for oxygen was also found at 20% RH. However, the effect of the CNC loading at this % RH was also the most significant, which points out that the hydrophilic and rigid nature of the CNCs and the lower crystallinity of the copolymer matrix, increased the free volume, thus facilitating gas diffusion. 

Although, in general nanocelluloses, when used as fillers, have been reported to improve the gas barrier properties of polymers [114,115], the fact that the polymer used in this study is already a very high-gas-barrier material explains the reduction observed [116]. Syverud et al. [117] reported OP values of 4.3–5.8 × 10^−20^ m^3^·m·m^−2^·Pa^−1^·s^−^^1^ for MFC films, whereas Nair et al. [118] reported OP values of 6.9 × 10^−21^ and 0.1–1.2 × 10^−21^ m^3^·m·m^−2^·Pa^−1^·s^−1^ for CNCs and EVOH films, respectively. In addition, it should be noted that the introduction of the CNCs in the experiments reported here was found to lead to a lower crystallinity for the copolymer, factor that is known to reduce permeability by increasing free volume and reducing tortuosity [119,120]. Similar observations have been previously reported after addition of MFC to EVOH, which led to a decrease in WVP due to changes in morphology and crystallinity [84]. The same observation was also found by Petersson et al. [121], who showed a reduction in OP when microcrystalline cellulose (MCC) was added to PLA due to a decrease in the degree of crystallinity.

## 4. Conclusions

The present study demonstrated the potential of the electrospinning process to obtain a new high-gas-barrier transparent fiber-based EVOH_44_ film. This material shows barrier properties somewhat lower than those of the same copolymer processed by other conventional processing technologies. The incorporation of CNCs increased the thermal and mechanical resistance of the fiber-based EVOH_44_ film, unfortunately for contents not exciding of 0.5 wt %, and exhibiting optimal balanced properties at 0.1 wt %. In this case, hybrid bio-/non-bio nanocomposites can be obtained with enhanced rigidity but reduced flexibility and slightly lower barrier properties. Still these hybrid nanocomposites offer the advantage of remaining in the high-barrier regime. The lower annealing temperature required for the EVOH_44_ copolymer and its nanocomposites with CNCs, enable them to be used as barrier interlayers, compatible with many more polymers and biopolymers than their higher vinyl-alcohol content homologous copolymers. Future work will deal with the application of these novel barrier materials as very thin interlayers in compostable multilayer systems, to ascertain the overall barrier reinforcement and physicochemical properties, and also their biodegradability under industrial and home composting conditions.

## Figures and Tables

**Table 1:** Properties of the poly(ethylene-*co*-vinyl alcohol) with 44 mol % of ethylene (EVOH_44_) solution and suspensions, with cellulose nanocrystals (CNCs), and mean diameter of their corresponding electrospun fibers and beaded regions.

Sample	Viscosity (cP)	Surface Tension (mN/m)	Conductivity (µS/cm)	Mean Fiber Diameter (nm)	Mean Diameter of Beaded Regions (µm)
EVOH_44_	71.9 ± 1.2 ^a^	23.8 ± 0.1 ^a^	11.63 ± 0.03 ^a^	410.0 ± 128.0 ^a^	1.1 ± 0.2 ^a^
EVOH_44_ + 0.1 wt % CNCs	74.3 ± 0.8 ^b^	24.1 ± 0.4 ^a,b^	11.80 ± 0.02 ^a,b^	410.4 ± 99.4 ^a^	1.3 ± 0.3 ^a,b^
EVOH_44_ + 0.5 wt % CNCs	77.4 ± 1.1 ^c^	24.3 ± 0.7 ^a,b^	12.00 ± 0.05 ^b,c^	501.7 ± 79.6 ^a^	1.5 ± 0.2 ^a,b^
EVOH_44_ + 1 wt % CNCs	80.1 ± 0.7 ^d^	24.9 ± 0.5 ^b^	12.10 ± 0.03 ^c^	592.4 ± 102.9 ^a^	1.8 ± 0.1 ^b^

^a–d^ Different letters in the same column mean significant difference among the samples (*p* < 0.05).

**Table 2:** Thermal properties of the electrospun poly(ethylene-*co*-vinyl alcohol) with 44 mol % of ethylene (EVOH_44_) fibers with and without cellulose nanocrystals (CNCs) in terms of: glass transition temperature (T_g_), melting temperature (T_m_), enthalpy of melting (ΔH_m_) and crystallinity (%X_c_), crystallization temperature (T_c_) and enthalpy of crystallization (ΔH_c_).

Sample	First Heating			Cooling	
	T_g_ (°C)	T_m_ (°C)	ΔH_m_ (J/g) (%X_c_)	T_c_ (°C)	ΔH_c_ (J/g)
EVOH_44_	40.6 ± 0.3 ^a^	163.9 ± 1.2 ^a^	60.1 ± 2.1 ^a^(27.0)	145.4 ± 0.8 ^a^	61.8 ± 2.1 ^a^
EVOH_44_ + 0.1 wt % CNCs	44.0 ± 0.5 ^b^	160.1 ± 2.2 ^b^	52.0 ± 1.0 ^b^(23.4)	137.2 ± 0.3 ^b^	50.1 ± 1.1 ^b^
EVOH_44_ + 0.5 wt % CNCs	42.5 ± 0.7 ^c^	157.2 ± 1.4 ^b^	53.4 ± 2.4 ^b^(24.1)	135.3 ± 1.0 ^c^	44.2 ± 1.5 ^c^
EVOH_44_ + 1.0 wt % CNCs	41.7 ± 0.2 ^c^	156.8 ± 1.6 ^b^	50.1 ± 1.1 ^b^(22.8)	133.2 ± 0.5 ^d^	38.5 ± 1.4 ^d^

^a–d^ Different letters in the same column mean significant difference among the samples (*p* < 0.05).

**Table 3:** Optical and color properties of the annealed electrospun films of poly(ethylene-*co*-vinyl alcohol) with 44 mol % of ethylene (EVOH_44_) with and without cellulose nanocrystals (CNCs).

Film	a*	b*	L*	ΔE*	T	O
EVOH_44_	2.81 ± 0.02 ^a^	−4.94 ± 0.04 ^a^	91.04 ± 0.05 ^a^	-	7.87 ± 0.04 ^a^	0.004 ± 0.001 ^a^
EVOH_44_ + 0.1 wt % CNCs	2.79 ± 0.03 ^a^	−4.69 ± 0.03 ^b^	91.28 ± 0.03 ^b^	0.35 ± 0.03 ^a^	7.70 ± 0.03 ^a^	0.003 ± 0.002 ^a^
EVOH_44_ + 0.5 wt % CNCs	2.81 ± 0.01 ^a^	−5.02 ± 0.05 ^a,c^	90.92 ± 0.04 ^a^	0.14 ± 0.04 ^b^	4.64 ± 0.02 ^b^	0.002 ± 0.001 ^a^
EVOH_44_ + 1.0 wt % CNCs	2.84 ± 0.02 ^a^	−5.10 ± 0.04 ^c^	90.84 ± 0.04 ^a^	0.26 ± 0.03 ^a,b^	3.61 ± 0.03 ^c^	0.002 ± 0.001 ^a^

a*: red/green coordinates (+a red, −a green); b*: yellow/blue coordinates (+b yellow, −b blue); L*: Luminosity (+L luminous, −L dark); ΔE*: color differences; T: transparency; O: opacity. ^a–c^ Different letters in the same column mean significant difference among the samples (*p* < 0.05).

**Table 4:** Thermogravimetric analysis (TGA) main parameters of the annealed electrospun films of poly(ethylene-*co*-vinyl alcohol) with 44 mol % of ethylene (EVOH_44_) with and without cellulose nanocrystals (CNCs) in terms of: onset temperature of degradation (T_5%_), degradation temperature (T_deg_), mass loss at T_deg_, and residual mass at 800 °C.

Sample	T_5%_ (°C)	T_deg_ (°C)	Mass Loss at T_deg_ (%)	Residual Mass (%)
CNCs	215.6 ± 0.4 ^a^	288.9 ± 0.5 ^a^	41.3 ± 0.2 ^a^	5.8 ± 0.8 ^a^
EVOH_44_	306.8 ± 0.9 ^b^	359.7 ± 0.7 ^b^	48.5 ± 0.3 ^b^	0.1 ± 0.1 ^b^
EVOH_44_ + 0.1 wt % CNCs	306.9 ± 0.7 ^b^	377.4 ± 1.4 ^c^	60.0 ± 0.5 ^c^	0.1 ± 0.1 ^b^
EVOH_44_ + 0.5 wt % CNCs	314.9 ± 1.5 ^c^	372.6 ± 1.1 ^d^	59.6 ± 0.8 ^c^	0.1 ± 0.1 ^b^
EVOH_44_ + 1.0 wt % CNCs	331.2 ± 0.3 ^d^	383.6 ± 1.4 ^e^	59.7 ± 0.4 ^c^	0.1 ± 0.1 ^b^

^a–d^ Different letters in the same column mean significant difference among the samples (*p* < 0.05).

**Table 5:** Mechanical properties of the annealed electrospun films of poly(ethylene-*co*-vinyl alcohol) with 44 mol % of ethylene (EVOH_44_) with and without cellulose nanocrystals (CNCs) in terms of: tensile modulus (E), tensile strength at yield (σ_y_), elongation at break (ε_b_), and toughness (T).

Film	E	$\sigma_{y}\;(\mathrm{MPa})$	$\varepsilon_{b}\;(\%)$	T (mJ/m^3^)
EVOH_44_	4699 ± 350 ^a^	38.7 ± 5.2 ^a^	10.4 ± 3.6 ^a^	3.2 ± 1.4 ^a^
EVOH_44_ + 0.1 wt % CNCs	6473 ± 257 ^b^	39.2± 8.6 ^a,b^	0.8 ± 0.5 ^b^	0.2 ± 0.1 ^b^
EVOH_44_ + 0.5 wt % CNCs	6022 ± 584 ^b^	30.2 ± 8.3 ^a,b^	0.7 ± 0.2 ^b^	0.2 ± 0.1 ^b^
EVOH_44_ + 1.0 wt % CNCs	4135 ± 399 ^a^	25.2 ± 7.7 ^b^	1.6 ± 0.7 ^b^	0.3 ± 0.1 ^b^

^a,b^ Different letters in the same column mean significant difference among the samples (*p* < 0.05).

**Table 6:** Values of water vapor permeability (WVP), and oxygen permeability (OP) of the annealed electrospun films of poly(ethylene-*co*-vinyl alcohol) with 44 mol % of ethylene (EVOH_44_) with and without cellulose nanocrystals (CNCs).

Film	WVP × 10^14^ (kg·m·m^−2^·Pa^−1^·s^−^^1^)	OP × 10^20^ (m^3^·m·m^−2^·Pa^−1^·s^−1^)		
		0% RH	20% RH	80% RH
EVOH_44_	1.6 ± 0.4 ^a^	1.1 ± 0.2 ^a^	0.10 ± 0.04 ^a^	4.7 ± 0.3 ^a^
EVOH_44_ + 0.1 wt % CNCs	3.7 ± 0.3 ^b^	1.4 ± 0.1 ^a^	0.38 ± 0.01 ^b^	8.3 ± 0.2 ^b^
EVOH_44_ + 0.5 wt % CNCs	4.1 ± 0.9 ^b,c^	2.7 ± 0.2 ^b^	0.53 ± 0.03 ^b,c^	8.8 ± 0.2 ^b^
EVOH_44_ + 1.0 wt % CNCs	4.5 ± 0.1 ^c^	2.3 ± 0.4 ^b^	0.61 ± 0.01 ^c^	8.7 ± 0.2 ^b^

^a–c^ Different letters in the same column mean significant difference among the samples (*p* < 0.05).
